# Validating the Predictions of a Dynamic Transmission Model Using Real-World Data from a Universal Varicella Vaccination Program in Germany

**DOI:** 10.3390/jmahp13020020

**Published:** 2025-05-06

**Authors:** Iwona Żerda, Tomasz Stanisz, Tomasz Fundament, Filip Chełmikowski, Wioletta Kłębczyk, Michał Pochopień, Emilie Clay, Samuel Aballéa, Mondher Toumi

**Affiliations:** 1 Clever-Access, Wadowicka 8a, 30-415 Krakow, Poland; 2 Complex Systems Theory Department, Institute of Nuclear Physics, Polish Academy of Sciences, Radzikowskiego 152, 31-342 Krakow, Poland; 3 Clever-Access, 53 Avenue Montaigne, 75008 Paris, France; 4 InovIntell, 3023GJ Rotterdam, Zuid-Holland, The Netherlands; 5 InovIntell, 215 rue du Faubourg St Honoré, 75008 Paris, France; 6 CEReSS/UR3279—Health Services Research and Quality of Life Center, Aix-Marseille University, 13385 Marseille, France

**Keywords:** epidemic modeling, vaccination impact modeling, dynamic transmission model, infectious disease dynamics, varicella, chickenpox

## Abstract

Dynamic transmission models (DTMs) have been used to estimate various aspects of the public health impact of varicella vaccination programs. The aim of this study was to validate the predictions of a DTM—developed using the typical approach to varicella modeling—using real-world data from a country with a long-term universal varicella vaccination (UVV) program and to assess the sensitivity of the predictions to changes in model input parameters. A compartmental, age-stratified DTM was developed using the settings corresponding to the existing UVV program in Germany. The model-predicted total number of varicella cases followed the same trend as observed in the reported data. The agreement between the simulations’ results and the data was the highest for the age group most exposed to varicella (0–5 years old), while for other age groups, a decline in accuracy was observed. Sensitivity analyses identified the input parameters having a crucial impact on the model’s long-term predictions. The results supported the reliability of the DTM for assessing the impact of varicella vaccination programs over the first decades after their introduction and provided an insight into how certain parameters and assumptions influence the model output and thus require careful evaluation in the studies of future varicella vaccination programs.

## 1. Introduction

Infectious disease epidemiology is influenced by a multitude of factors, including pathogen characteristics, human immune system response, social contact patterns, and population mobility. Dynamic transmission modeling serves as a mathematical framework designed to capture the evolving dynamics of infectious disease epidemiology under various scenarios. These scenarios may include the introduction of preventive programs or changes in age structure and social contact patterns. Dynamic transmission models (DTMs) are widely recognized for their usefulness in economic evaluations of vaccination programs [1,2,3]. Health technology assessment (HTA) bodies and health agencies have identified DTMs as the appropriate methods for the design and assessment of the potential public health impact of vaccination interventions [4,5,6,7,8,9]. While DTMs are typically calibrated to pre-vaccination era data and often used for predicting the future impact of vaccination programs, surveillance systems are generally implemented after the introduction of such programs to monitor the actual impact on targeted infectious diseases. It is often the case that model predictions made during the assessment of vaccination programs are not validated with real-world data from these systems in the post-vaccination era, even for diseases with long-standing vaccines.

Varicella, commonly known as chickenpox, is an infectious disease caused by the varicella zoster virus (VZV), characterized by a pruritic vesicular rash that typically appears 10 to 21 days after exposure 15. The disease begins with a mild prodrome of fever and malaise, followed by the eruption of lesions that progress from macules to vesicles before crusting over [10]. Transmission occurs primarily through the airborne route, with infectious particles released from skin lesions or respiratory secretions [11,12]. The health burden associated with varicella includes complications such as bacterial superinfection of skin lesions, pneumonia, and neurological issues [10,13]. Risk factors for severe disease include immunosuppression, age (with higher severity in adults), and pregnancy [10]. The incubation period for VZV ranges from 10 to 21 days; the infective period lasts until all lesions have crusted over (typically 4–7 days after the onset of rash) [12].

Varicella has a significant economic impact; for example, in the absence of UVV, the total annual costs generated by varicella for Europe in 2018 have been estimated to over EUR 660 million (in a large part related to caregiver work loss) [14]. The importance of effective prevention strategies can be demonstrated with the example of the United States, where the net societal savings for 25 years of UVV program implementation were estimated to USD 23.4 billion [15].

Live attenuated viral vaccine against varicella was developed in the early 1970s. The vaccine, available as monovalent or combined with other vaccines like measles, mumps, and rubella, has demonstrated good tolerance. The estimates of the vaccine effectiveness range from 55% to 87% for one dose and from 84% to 98% for two doses [16].

To address the public health impact of varicella, many countries, including Germany, have implemented vaccination programs. Germany introduced universal childhood varicella vaccination already in 2004, initially as a single dose, which was later expanded to a two-dose series in 2009. This was the first nationwide vaccination program against varicella in Europe. To assess the vaccination program’s impact, Germany established a sentinel surveillance system to monitor varicella cases through general and pediatric practices nationwide [17]. The data were collected from the beginning of 2005 to the end of 2017. In March 2013, the varicella countrywide notification system was introduced. Consequently, the effects of the vaccination program have been documented since its inception, although it can be expected that the recorded numbers of infection cases are significantly lower than the actual ones, due to underreporting, which is known to occur with varicella [18,19,20].

DTMs have been utilized in several studies on the impact of varicella vaccines in Germany [21,22,23,24,25] for purposes like assessing the cost-effectiveness of planned vaccination strategies or addressing concerns about potential increases in varicella infection age and herpes zoster incidence following the implementation of universal varicella vaccination (UVV) in young children. To replicate the local epidemiology of varicella, these models were calibrated to the data observed in the pre-vaccination era. As most of the modeling studies were conducted before or shortly after the varicella vaccination program was introduced, their results could not be validated against the observed varicella cases. For the others, validation against observed data was not the purpose of the analysis. Horn et al. (2018) [25] analyzed how the combination of various vaccination programs and demographic dynamics will affect the epidemiology of varicella and herpes zoster in Germany over the timeframe of 50 years. Focusing on the methodological aspects of varicella and herpes zoster modeling, the study used the data from the vaccination period (up to 2012) to calculate the parameters for vaccination (efficacy, primary vaccine failure, and waning) and assess their impact. In Horn et al. (2016) [24], the model predictions were validated with the observed data up to 2009. The authors shortly stated that the model correctly reflects the short-term effects (i.e., a considerable reduction in varicella incidence among children younger than 10 years of age) as they were documented in the established sentinel surveillance system; however, no quantitative measures of this validation were presented. Thus, the validation of model predictions to observed data for existing models of varicella vaccination in Germany has been performed in a limited scope—as seems to be the case with many model-based studies [26].

The literature has provided data on the social and epidemiological landscape of Germany prior to the introduction of varicella vaccination program, along with the documentation of program outcomes over the years. Despite limitations, such as varicella underreporting and potential regional heterogeneity in the vaccination program, these data present an opportunity for assessing vaccination modeling in this country, allowing one to build and validate the model using real-world observations whilst mitigating the impact of data gaps and model assumptions.

The objective of this study was to build a DTM (with the structure appropriate for modeling varicella) to compare its predictions with the real-world data on varicella epidemiology and the public health impact of varicella vaccination. Additionally, the study aimed to test how the model predictions are influenced by individual input parameters. By leveraging pre-vaccination data and information regarding the implementation of the vaccination program from 2005 to 2017, the model was adapted to the German UVV. The results generated by the model were compared to the reported impact of varicella vaccination on disease burden within the country. The model predictions were further explored beyond the existing vaccination program timeframe and across a range of model inputs to identify the key drivers of varicella modeling, with a view of informing future modeling studies. Through validation against empirical data and sensitivity analyses on key input parameters, this study aimed to understand the model’s ability to provide valuable insights into the impact of vaccination program on disease transmission dynamics.

## 2. The Model

**Figure 1:**
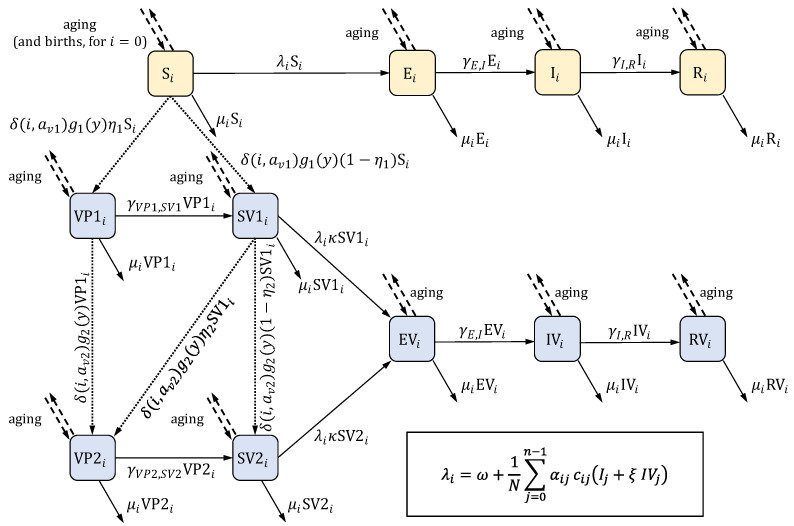
The structure of the model. The scheme presents the compartments (listed in Table 1) and the flows between them for a single age group (indexed by i). Compartments colored in yellow pertain to natural varicella in the absence of any protection from varicella vaccination; compartments colored in blue are related to vaccination. Solid arrows represent flows in continuous time, and their labels give the flow rates. Most of these rates can be written as $r\cdot X_{i}$, where r is a constant, and $X_{i}$ is a compartment; the exceptions are the rates involving the force of infection $\lambda_{i}$, which is given by the formula in the frame in the bottom right corner (in the formula, N denotes the total number of individuals in the population; the remaining parameters are listed in Table 2). Dashed arrows represent aging and, for the first age group, births, both of which occur in discrete time steps at the beginning of each year. Aging involves moving all individuals from one age group to the next age group (i.e., from compartments $X_{i}$ to $X_{i+1}$) for all but the last age group, from which individuals are removed from the model. Births occur only for the compartment $S_{0}$ (the “S” state in the first age group). Dotted arrows represent vaccination, which also takes place at the beginning of each year. Labels express the number of individuals moved between the compartments. δ() denotes the Kronecker delta function: $\delta \left(i,j\right)$ is equal to 1 when i=j and equal to 0 when i≠j. The description of the involved parameters is given in Table 2.

The model was a compartmental DTM—the studied population was divided into compartments (representing different health states relevant to infection transmission), and the changes in the system were represented by flows between the compartments, expressed in terms of differential equations. Its structure was based on a SEIR model (Susceptible—Exposed—Infectious—Recovered), extended by compartments representing vaccinated individuals. Models of this type were routinely used in modeling the transmission of varicella [27,28,29,30,31]. The list of model states is given in Table 1. The model covered the population aged from 0 to 99 years (individuals leave the model when they reach 100 years of age). The model took the population’s age structure into consideration in a way typical for the so-called realistic age-structured models [27,28]: the population was divided into annual age groups, and aging was applied in discrete time steps at the beginning of each year. Births and vaccination were also incorporated in these steps. During each simulated year, the evolution of the system was driven by ordinary differential equations (ODEs) to model the processes of becoming infected, recovering from infection, immunity waning, and mortality. The force of infection depended on the number of infected individuals and on the “case importation constant”—a parameter representing the contribution to the infection dynamics stemming from the infection cases imported due to migration and travel. The model allowed for gradual changes in the population age structure resulting from changes in the number of births in consecutive years (mortality in each age group was assumed to be constant over time). The set of compartments consisted of two categories: those corresponding to natural varicella infection in the absence of any protection from varicella vaccination and those related to vaccination. It was assumed that individuals who acquired vaccine-induced immunity are invulnerable to infection, although vaccine-induced immunity might not be lifelong, as the model considered immunity waning for each vaccine dose. The model considered the differences in infectiousness and susceptibility between the vaccinated and unvaccinated individuals. The structure of the model is demonstrated for a single age in Figure 1. The equations are presented in Appendix A.2.

The model parameters for demography, contact patterns, natural varicella infection, and vaccination (including effectiveness and waning) were derived from the literature. The transmission of the virus was modeled using the age-specific number of infectious subjects, contact matrices, and the transmissibility of varicella virus per contact. The age-specific transmissibility was calibrated to ensure that the disease prevalence predicted at equilibrium in the ODE system without vaccination closely matched the empirically observed prevalence in the pre-vaccination era (details are provided in Appendix A.3). The initial state of the model, representing the pre-vaccination period, was defined as this equilibrium state of the ODE system.

The ODE system considered continuous time for all flows (see Appendix A.3 for the relevant equations), while the birth and aging processes in the model operated in discrete time. Consequently, the equilibrium states of the model and the ODE system used in the calibration may slightly differ. To stabilize the total population, the model was initialized after a 100-year burn-in period, representing the maximum lifespan. Any simulations, including those with vaccination included, were conducted after this period.

The pathogen causing varicella is also responsible for herpes zoster; therefore, these two diseases are often modeled jointly [24,25,31,32,33]. Nevertheless, the influence of herpes zoster cases on the force of varicella infection can be considered negligible [30,32], while in the model discussed in this work, only the number of varicella cases is of interest. Hence, for simplification, compartments referring to herpes zoster are not included in the model (similar as in Brisson et al. (2000) [30]).

## 3. Data

The model parameters were used to characterize infection duration and prevalence in pre-vaccination era, demography (population age structure, births, and mortality), social contact patterns, and vaccination (vaccine effectiveness, vaccination coverage, immunity waning rate, etc.). To assess the performance of the model, empirical data on the reported number of varicella cases in post-vaccination era were used. The main sources of the data are presented below.

### 3.1. Natural Varicella Infection, Demography, and Social Contact Patterns

Information on the natural varicella infection (including the duration of the latent period and of the infectious period) is widely available, for example, in Heininger and Seward, 2006 [34] or in the CDC resources [35]. The estimated numbers of births for each year in the modeled period were taken from United Nations data and forecasts [36], along with age-dependent mortality rates (assumed to be time-independent). Social contact matrix in Germany between May 2005 and September 2006 (i.e., shortly after the vaccination introduction) is reported in Mossong et al. (2008) [37]. The number of contacts in the matrix was presented for the age groups, while the model considers single ages. Consequently, the contact matrix was appropriately transformed before being supplied to the model’s input (see Appendix A.4 for details).

### 3.2. Varicella Prevalence and Incidence

Varicella prevalence before the introduction of the vaccination program (i.e., before 2004) was estimated based on the seroprevalence data. Data for children and adolescents (1–17 years old) were taken from Wiese-Posselt et al. (2017) [38], while data for older age groups were derived from Bollaerts et al. (2017) [39]. To account for the increase in varicella seroprevalence with age, which was not fully reflected in the empirical data, a smooth, monotonic function was fitted to the pooled data and used for prevalence estimation. Under the assumption of permanent infection-induced immunity, the increments of seroprevalence for consecutive age groups can be related to disease incidence and, consequently, to prevalence.

Infants born to varicella-immune mothers receive maternal antibodies against varicella, providing initial protection at birth. However, this maternal protection quickly wanes, leaving infants vulnerable to varicella infection within their first year of life. To account for the effect of maternal protection, data on seroprevalence for infants (under one year of age) from Wutzler et al. (2001) [40] were utilized to adjust the estimation of prevalence for the relevant age groups. Details of the prevalence estimation procedure are outlined in Appendix A.1.

Prior to 2004, varicella was not a nationally notifiable disease in Germany [17], resulting in the absence of data on pre-vaccination varicella incidence. Furthermore, published data on post-vaccination varicella incidence has been scattered and, in some sources, likely severely underreported [18,19,20]. The Robert Koch Institute, a German federal government agency, collected and published data from a varicella-specific sentinel surveillance program [41]. Another source of information was the German statutory health insurance system [42]. Since 2014, annual reports on varicella incidence have been available in the federal government’s online health reporting database (Gesundheitsberichterstattung) [43]. In Moek and Siedler, 2023 [17], these three sources were presented in a unified manner, providing the empirical data on varicella incidence used in this study.

### 3.3. Vaccination

In 2004, one-dose UVV was implemented in Germany for all infants aged 11 to 14 months. In 2009, this was extended to a two-dose regimen, where the second dose is recommended at 15 to 23 months of age [44]. Since the model implemented vaccination in discrete, annual time steps, the first dose uptake was assumed to occur at the age of 1 year, and the second dose was assigned to the age of 2 years. The effectiveness of the vaccine was estimated according to Liese et al. (2013) [45] and Siedler et al. (2016) [46].

The estimation of vaccination coverage was based on data published by the Robert Koch Institute [47]. The data were collected during the school entry examinations, which are conducted in Germany for children aged between 4 and 7 years. Coverage data for the first dose were available for the years 2008–2019, while data for the second dose were available for 2010–2019. To estimate the vaccine coverage at the time of vaccination, the reported coverage rates were adjusted backwards in time. Knowing that school entry examinations typically took place at the age of 5 [48,49], the adjustments were as follows:

- 4 years for the first dose (administered at the age of 1);
- 3 years for the second dose (administered at the age of 2).

The coverage rates stabilized from 2015 onwards, so the rates from 2019 were used for the years 2020 and later. The model did not account for the spatial distribution of the population and infection cases; thus, the yearly vaccination coverage rates were applied uniformly across the entire country.

Vaccine-induced immunity for both the first and second doses was assumed to wane at a constant rate. The waning rates were based on values from Horn, 2016 [24] and Horn, 2018 [25].

### 3.4. Other Parameters

The value of the case importation constant ω was based on Ouwens et al. (2015) [32] and Akpo et al. (2020) [33]. Parameters modifying infectiousness of breakthrough infections and the susceptibility of vaccinated individuals who are susceptible, denoted as ξ and κ, respectively, were derived from Brisson et al. (2000) [30].

**Figure 2:**
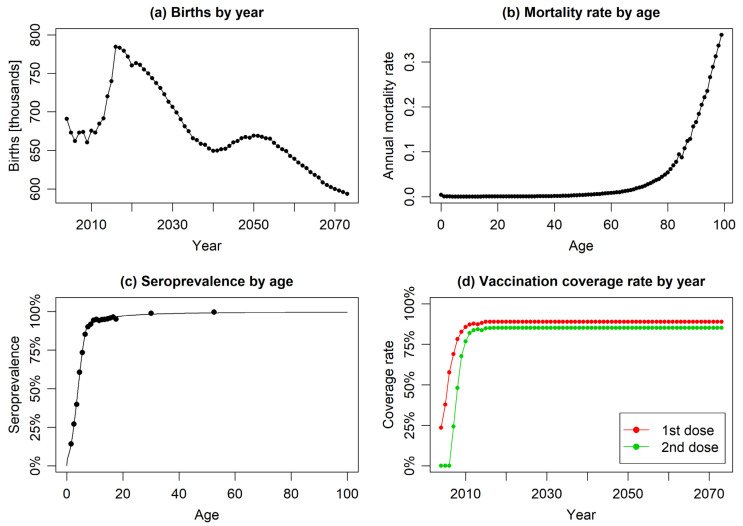
The year or age-specific model input parameters: (**a**) reported and forecasted annual numbers of births, (**b**) age-specific mortality rates, (**c**) empirical data on varicella seroprevalence by age (scatter plot) with fitted regression model (solid line), and (**d**) annual vaccination coverage rates for first and second doses (constant coverage assumed from 2017 onwards).

The values of the above-discussed input parameters of the model are presented collectively in Table 2 and in Figure 2.

**Table 2:** The input parameters used in the base case scenario. The last two variables, ${\hat{J}}_{i}$ and $\alpha_{ij}$, can be considered slightly different from the remaining ones as they are not “raw inputs”, but are derived from the input data (${\hat{J}}_{i}$ is estimated based on seroprevalence, and $\alpha_{ij}$ is computed in model calibration).

Parameter	Value	Description	Source
n	100	number of annual age strata (age groups); age groups are indexed by numbers 0, 1, 2,…, n−1	assumption
b(y)	see Figure 2	number of births in year y	UN data [36]
$\mu_{i}$	see Figure 2	mortality rate in age group i	UN data [36]
$\gamma_{E,I}$	$\frac{1}{14\;days}$	rate at which an exposed individual becomes infectious (assumed to be equal to $1/T_{latent}$, where $T_{latent}$ is the average duration of the latent period of the disease)	Heininger and Seward, 2006 [34], CDC [35]
$\gamma_{I,R}$	$\frac{1}{7\;days}$	rate at which an infectious individual recovers (assumed to be equal to $1/T_{infection}$, where $T_{infection}$ is the average duration of the infection)	Heininger and Seward, 2006 [34], CDC [35]
$a_{v1}$	1	age of vaccination with the first dose	assumption, Siedler and Arndt [44]
$a_{v2}$	2	age of vaccination with the second dose	assumption, Siedler and Arndt [44]
$g_{1}\left(y\right)$	see Figure 2	coverage of the first dose of vaccination in year y	RKI reports [47], information about school entry age [48,49]
$g_{2}\left(y\right)$	see Figure 2	coverage of the second dose of vaccination in year y	RKI reports [47], information about school entry age [48,49]
$\eta_{1}$	81.9%	effectiveness of the first vaccine dose	Liese et al. (2013) [45] and Siedler et al. (2016) [46]
$\eta_{2}$	94.4%	effectiveness of the second vaccine dose	Siedler et al. (2016) [46]
ξ	0.5	rate of infectiousness of breakthrough infections (in vaccinated individuals) compared to natural infections (in unvaccinated individuals)	Brisson et al. (2000) [30]
κ	0.73	rate of susceptibility of vaccinated individuals who are susceptible for the infection compared to unvaccinated individuals	Brisson et al. (2000) [30]
$\gamma_{VP1,SV1}$	$\frac{1}{40\;years}$	waning rate of the immunity induced by single-dose vaccination	assumption, Horn, 2016 [24] and Horn, 2018 [25]
$\gamma_{VP2,SV2}$	$\frac{1}{80\;years}$	waning rate of the immunity induced by two-dose vaccination	assumption, Horn, 2016 [24] and Horn, 2018 [25]
$c_{ij}$	age-specific values in the range between 1.86 and 73.52	parameter describing social contact rate: $c_{ij}/N$ is the average number of contacts made by a specific individual from age group i with a specific individual from age group j per unit time (N) is the total population size)	computed based on data derived from Mossong et al. (2008) [37]; see Appendix A.4
ω	$0.001\frac{1}{year}$	case importation constant representing the contribution of imported varicella cases to the force of infection ($\lambda_{i}$)	assumption, Ouwens et al. (2015) [32] and Akpo et al. (2020) [33]
${\hat{J}}_{i}$	age-specific values in the range between $9.27\cdot 10^{-7}$ and $3.16\cdot 10^{-3}$	disease prevalence observed within age group i, used for calibrating the values of $\alpha_{ij}$	computed based on seroprevalence derived from Wiese-Posselt et al. (2017) [38], Bollaerts et al. (2017) [39], and Wutzler et al. (2001) [40]; see Appendix A.1
$\alpha_{ij}$	age-specific values in the range between 0.033 and 0.429	transmissibility of the virus representing the probability that contact between an infectious individual from age group j and a susceptible individual from age group i results in infection transmission	calibrated; see Appendix A.3

## 4. Results

### 4.1. Validation of Model Predictions Against Reported Data

**Figure 3:**
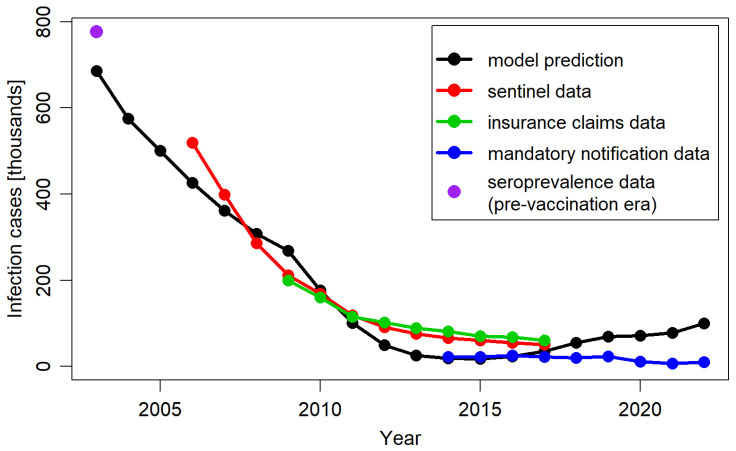
Total number of varicella cases per year, predicted by the model, extracted from Moek and Siedler, 2023 [17] (sentinel data, insurance claims data, and mandatory notification data) and estimated based on seroprevalence data in the pre-vaccination period.

Figure 3 presents the annual total number of varicella cases predicted by the model over a 19-year post-vaccination period, alongside the corresponding annual cases derived from empirical data. The model-predicted decrease in varicella cases aligned well with trends observed based on available data sources. The levels of inconsistency between the model predictions and the empirical data were similar to those identified between the empirical data sources, which were present likely due to variations in methodology and population selection.

**Figure 4:**
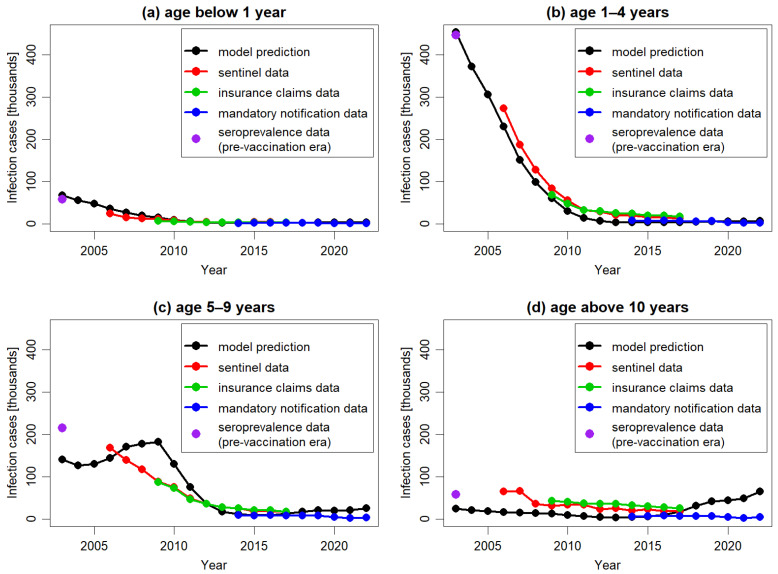
The number of varicella cases per year, predicted by the model and extracted from Moek and Siedler, 2023 [17] (sentinel data, insurance claims data, and mandatory notification data) and estimated based on seroprevalence data in the pre-vaccination period, for different age groups: 0 years (**a**), 1–4 years (**b**), 5–9 years (**c**), and above 10 years (**d**). To facilitate comparison, all plots are in the same scale.

In Figure 4 the model predictions and corresponding empirical data regarding the impact of vaccination were presented across different age groups. In line with observed data, the model predicted a long-term decline in varicella cases across all age groups compared to the pre-vaccination period. The model’s outcomes most closely aligned with empirical data for the age group 1–4 years, which contributed the most to the total number of varicella cases in the population. The model accurately replicated the trend of decline reported for this age group from 2007 onwards, although consistently presenting lower numbers of cases than those reported in the sentinel system and insurance claims data. The correct replication of the case numbers estimated for the pre-vaccination period and reported in the mandatory notification system suggested that the observed discrepancy for the other data sources might be related to different reporting patterns.

For older age groups, the model’s predictions were less accurate. Specifically, for the age group 5–9 years, the model predicted an increase in the number of cases between 2004 and 2009, followed by a decline in subsequent years, while for the older age group the model predicted an increase in the number of cases from 2015 onwards. None of these increases were observed in the empirical data. The predicted increases were the result of the dynamics of transmission simulated in DTMs. In particular, any decrease in the number of cases triggers a cascade of consequences. This includes a decline in infection transmission across the entire population, which in turn leads to a reduction in the number of cases and an increase in the number of susceptible subjects. If the infection is not eradicated and the virus continues to circulate, the number of susceptible individuals will eventually reach a threshold high enough to spark a new outbreak, resulting in an increase in cases. These effects, although occurring at different times, were visible in both the 5–9 years age group and the 10 years and older age.

Furthermore, the study results indicated that the model predictions underestimated the number of varicella cases in the pre-vaccination period. When stratified by age groups, the largest discrepancies were observed in age groups that contributed less to the total number of varicella cases, while the outcomes for the 1–4 years age group were consistent with observed data. These discrepancies in older age groups likely were the results of the differences between the ODE system used in the calibration process and the one used in model simulation. Ideally, both systems should be identical to ensure accurate predictions. In the model, to ensure accurate aging (specifically, to increase the age of all simulated subjects every year), this process was considered in discrete time. Similarly, births and vaccinating processes were operated in discrete time. This approach prevented the simulated population from stabilizing, causing yearly fluctuations. In a model of the discussed type, disease prevalence changes during the course of each year, even when input parameters are fixed, and the model has time to equilibrate. Therefore, the calibration of such a model—which attempts to find input parameters allowing one to reproduce the observed prevalence—requires running simulations with many different parameter configurations and selecting the configuration for which the annually averaged predicted prevalence is the closest to the target prevalence. If multiple parameters are calibrated, such an approach becomes infeasible. As the varicella epidemiology is age-dependent, at least four age groups, and thus four parameters, are typically considered for calibration. However, the inclusion of more age groups results in greater accuracy. This prevented the use of the mentioned computational procedure in model calibration. Consequently, in the calibration process, a system of equations was used that treated births and aging as continuous processes. This simplified the computations considerably. However, the calibration system differed slightly from the model simulation system, which affected the model accuracy—this was most pronounced in older age groups. Despite this, the differences in the model-predicted and observed varicella cases were relatively minor when considered in the context of the total number of cases. Consequently, the study results indicated that although the model effectively captured the general epidemiological characteristics of varicella in Germany, its predictions for specific age groups, particularly those less affected by the infection, should be interpreted with caution.

### 4.2. Identification of the Model Key Drivers

**Figure 5:**
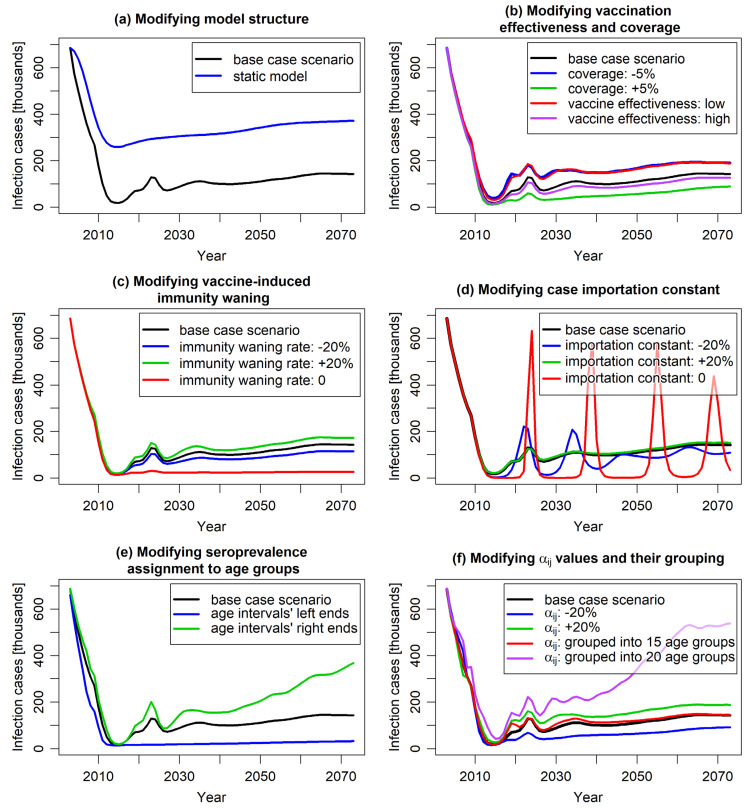
The predicted number of varicella cases over a 70-year timespan in the base case scenario and across scenarios modifying specific model parameters. Plot (**a**) illustrates the comparison of the base case scenario with a static-model scenario in which the force of infection was fixed at pre-vaccination level. Plots (**b**–**d**) illustrate the impact of varying vaccine effectiveness and coverage (**b**), the vaccine-induced immunity waning rates (**c**), and the case importation constant (**d**). Plot (**e**) presents the implications of different interpretation of seroprevalence data alignment (whether the reported data are interpreted as pertaining to the ends or centers of relevant age intervals). Plot (**f**) demonstrates the effect of altering the age-specific probability of varicella transmission per contact $\alpha_{ij}$—directly or by changing the age grouping in calibration (the groupings are specified in the text).

One-way sensitivity analyses were performed to evaluate the impact of variations in model parameters on model predictions and their validation against the empirical data. The analyses included model structural parameters and the most uncertain data inputs, such as varicella epidemiology, vaccine efficacy, coverage, and waning rate. For most scenarios, the results remained relatively consistent within the timeframe of the empirical data. To assess their implications over an extended period beyond this timeframe, a long-term perspective spanning 70 years was adopted. The results are presented in Figure 5.

The results of most of the studied scenarios were comparable to those of the base case scenario in terms of the predicted number of infection cases, aligning with the empirical data observed within the 19 years after the vaccination introduction. However, differences between various scenarios started to emerge on longer time scales.

Comparing the results of the “static model”, which assumed a constant force of infection from the pre-vaccination period, with the results of corresponding dynamic model (Figure 5a), confirmed that static modeling underestimated the impact of vaccination. Unlike dynamic models, static models neglect the indirect effects of vaccination, focusing solely on its direct impact on vaccinated individuals [7]. As a result, while both static and dynamic models predicted reduced varicella cases post-vaccination, the static model showed a noticeably slower decline that persisted in the long term. Beyond 19 years post-vaccination, both models indicated a slight increase in cases due to waning vaccine protection. Given varicella’s high contagiousness, UVV was expected to significantly reduce the viral reservoir, thereby lowering the risk of infection for both vaccinated and unvaccinated individuals. This indirect effect was considered by the dynamic modeling approach. The study results suggested that the underestimation of vaccination impact by static models compared to dynamic model may be substantial.

Varying the vaccine effectiveness (to 71.5% and 86.4% for the first dose and to 86.6% and 97.3% for the second dose) and the vaccination coverage (by ±5%) did not significantly alter predictions for the first 15 years post-vaccination. Similar to the base case scenario, a sharp decrease in the number of infection cases was observed (Figure 5b). However, after this initial period, the number of cases stabilized at a level clearly dependent on these parameters, highlighting the importance of accurate evaluation of these parameters for long-term prognosis.

The model predictions for varying waning of vaccine-induced immunity showed similar behavior (Figure 5c). Changing the waning rates by 20% roughly maintained the shape of the curve representing the predicted number of cases over time, with slight variations in long-term values. An exception was the scenario assuming no waning of vaccine-induced immunity. In this case, the initial sharp decrease in infection incidence was permanent, resulting in only sporadic cases observed in the long term. This outcome is intuitive as the absence of immunity waning is quantitatively different from scenarios with varying waning rates.

An important factor shaping the model results was the case importation constant (Figure 5d). This constant was incorporated to capture the impact of human mobility, such as migration and travel, on varicella incidence. In the short term, it had minimal impact on model predictions. However, over the long term, it proved pivotal in modeling the timing and magnitude of varicella outbreaks. The case importation constant represented the continuous influx of infectious cases into the population, independent of the varicella vaccination program. Increasing its value stabilized infection rates, flattening the annual incidence curve. Conversely, reducing its value led to sharper declines in cases and longer periods with minimal virus circulation, resulting in strong epidemic waves once the reservoir of susceptible individuals became infectious. In an extreme case—when the constant was set to 0—the predicted number of cases exhibited slowly decaying oscillations between near-zero and pre-vaccination levels, highlighting the importance of accounting for human migration in vaccination impact evaluations. It is worth noting that such oscillations are known to occur in compartmental models taking demography (births and deaths) into account [50]; their amplitude depends on the parameters describing the disease and the population dynamics. From a technical point of view, non-zero case importation constant keeps the force of infection above some threshold value (which can here be attributed to migration and travel) and damps the incidence oscillations by preventing the accumulation of individuals in the “susceptible” compartment. Notably, increasing the case importation constant above the base case value did not fundamentally change the model outcomes. This suggests a potential threshold for the constant that eliminates all significant oscillations arising from the model’s structure and dynamics.

Scenarios on calibration parameters also provided valuable insights. Due to the well-known underreporting of varicella in surveillance systems, disease modeling often relies on seroprevalence data, which are less affected by this issue. Seroprevalence data are typically reported for age intervals. In models requiring seroprevalence as an increasing function of age, these data are assigned to interval midpoints, with an assumed increasing curve between them. However, considering the unknown distribution of individuals within age groups, choosing the interval bounds (or intermediate values) might also be appropriate. Study results showed that for varicella seroprevalence, which rises sharply in younger age groups and flattens in older ages, this choice can significantly impact calibration outcomes and, consequently, the model results (Figure 5e).

Figure 5f demonstrates the impact of age grouping in the calibration process on model results. In the base case, each age was individually considered (resulting in 100 independent $\alpha_{ij}$ parameters), ensuring precise modeling of varicella transmissibility per contact. Other models often group ages, reducing the number of calibrated parameters but potentially compromising accuracy depending on the width and arrangement of these groups. Figure 5f compares outcomes for a scenario with 15 age groups (10 1-year groups from 0 to 9 years, followed by groups 10–14, 15–19, 20–24, 25–59, and 60–99 years) and a scenario with 20 5-year age groups. The 15-group scenario closely matched the base case, indicating it sufficiently captured age-related transmissibility variations despite fewer age groups. In contrast, the 20-group scenario deviated significantly from the base case, particularly for children and adolescents, where $\alpha_{ij}$ variability was highest. This can be attributed to the insufficient resolution of the grouping in the age range most important from the perspective of infection transmission; the effect highlights that both the selection and number of age groups are crucial for model precision.

In parallel, varying transmissibility per contact without changing age structure of the parameter had marginal short-term and moderate long-term effects on model predictions (Figure 5f), suggesting that age dependence, rather than magnitude variation, is a critical driver of the model outcomes.

## 5. Discussion

This modeling study aimed to reproduce the public health impact of the UVV introduced in Germany in 2004 during the post-vaccination period, leveraging the latest real-world evidence and validated the model predictions against the program outcomes over 19 years of vaccination implementation. The study findings indicated that the number of varicella cases predicted by the DTM closely mirrored the trend in reported data following the UVV introduction. Despite the simplifications associated with the modeling technique and the limitations of data availability, the model demonstrated reliability in capturing the general characteristics of varicella transmission dynamics. These findings justify using dynamic transmission modeling as a standard tool for assessing the public health impact of vaccination programs, particularly in the context of varicella immunization.

While validating modeling results using post-vaccination empirical data is widely recommended [26,51], this practice is not commonly observed. Many DTMs have been developed in the time preceding the introduction of specific vaccines to predict future disease epidemiology after vaccination programs were implemented and to assess the public health impact of such programs. Typically, these models were calibrated using data observed in the pre-vaccination era [21,30,33,52]. Once a vaccination program has been established, real-world data are prioritized over simulation results, and model predictions were often not validated against empirical observations. In cases where DTMs were developed after vaccination had already been introduced, available post-vaccination data were frequently used to calibrate parameters such as vaccine effectiveness and waning rates, thereby enhancing prediction accuracy [24,53]. The utility of the empirical data in validating such models is limited. Consequently, validating DTM predictions for specific varicella vaccination program against real-world data from sources independent of those used in model setup provides valuable insights.

Varicella serves as a convenient example for the modeling of infectious disease due to its straightforward nature. In comparison, many other diseases present greater complexity in various aspects: COVID-19, with the emergence of new variants; meningococcal and pneumococcal infections, which involve serotype replacement; influenza and dengue, each with multiple serotypes; and chlamydia and HIV, transmitted through sexual contacts. Varicella, being transmitted directly between individuals, involving a single pathogen and following a uniform course, provides an excellent example for validation purposes since it allows to maximally simplify the model and to focus on the modeling of the dynamics of an infectious disease transmission in a changing population and valuate DTM utility in such applications.

The outcomes of this validation study confirm earlier findings regarding the dynamic modeling of varicella infection in both Germany and other countries with well-established, long-term varicella immunization programs. Notably, these models predict a substantial reduction in the varicella burden following the introduction of the vaccination programs across nearly all age groups, particularly those directly targeted by the vaccination campaigns. These projections have found empirical support in data collected through surveillance systems [17,54] during the post-vaccination period. Simultaneously, the incorporation of indirect effects within dynamic models often leads to predictions of increased disease incidence among older age groups [16,24,55,56,57]. Consequently, it was anticipated that the proportion of complications would rise, given that clinical presentations tend to be more severe in older individuals. These predictions, coupled with concerns about a potential increase in herpes zoster (HZ) incidence due to the elimination of virus circulation, have contributed to delays in the introduction of UVV in many countries. Despite the age shift in the disease predicted by modeling studies conducted across various countries, including the previous models for Germany, the data observed over years post-vaccination indicate declining overall incidence rates among adolescents and adults [54,58,59,60]. Similarly, surveillance data from Germany demonstrate reduced varicella incidence in all age groups [17] over a 19-year period post-vaccination; despite this, minor increases in incidence among individuals aged 10 years and over were predicted by the model under the study. The reasons for the discrepancy between the expected age shift in the disease and empirical observations remain unclear. Continuous surveillance will be essential to evaluate the necessity of catch-up vaccination in specific populations at risk.

A range of scenarios with varying parameters regarding vaccination and disease transmission was considered. A significantly better fit to reported observations was found for dynamic modeling predictions compared to the estimates based on static force of infection, which substantially underestimated the impact of the considered vaccination program. This aligns with the methodology of other models for varicella vaccination, which were consistently developed as dynamic models. Across all the scenarios examined, the implementation of UVV was consistently associated with a reduction in varicella incidence. Most of the scenarios predicted a similar reduction in varicella cases over a 19-year post-vaccination period while exhibiting significant differences between each other in terms of the public health impact of UVV in Germany projected over the later decades. Apart from vaccine effectiveness, immunity waning rate, and the assignment of seroprevalence data by age, parameters related to human mobility and to the dependence of disease transmission on age have been tested with respect to their influence on the model predictions. These parameters are rarely tested in publications using DTMs; however, they turned out to have a profound impact on the long-term model outcomes. These results highlight the importance of carefully selecting the modeling approach and the relevant input parameters to ensure the highest possible accuracy.

Some of the assumptions utilized in this study are subject to a certain level of uncertainty. This leads to several limitations of the model. Firstly, the model does not take herpes zoster (HZ) into account as HZ has a marginal impact on varicella transmission [30,32]. While such an approach is justified when the analysis is focused only on varicella cases (like in this study), it is inappropriate when the whole process of varicella-zoster-virus circulation is under investigation—then, a model with a more complex structure is required. Such detailed models can, for example, predict that UVV might lead to an increase in the burden of HZ due to an assumed pronounced exogenous boosting effect [30,56].

Additionally, vaccination coverage data in Germany were monitored during the medical examination conducted typically a year before school entry. As a result, the coverage data were reported with a delay of 3–6 years after the age when children were eligible for varicella vaccination, creating an information gap that might affect model estimates. Furthermore, the accuracy of the model is limited by its sensitivity to changes in seroprevalence data preprocessing and the presence of underreporting in validation data. The precision of model predictions is also affected by the approximate character of the equations used in calibration—which is particularly noticeable for older age groups.

Finally, unaccounted regional differences in varicella incidence and vaccine uptake across Germany may impact the model’s ability to predict varicella incidence trends post-vaccination accurately. Despite both variables having been reported inhomogeneous across different regions [44], it is expected that these discrepancies were likely driven by delays in vaccination program implementation (i.e., reimbursement policies) and variations in the quality of regional surveillance systems. It is expected that the influence of these factors on the model outcomes is limited.

In conclusion, despite its limitations, this study demonstrated the utility of dynamic transmission modeling in evaluating varicella vaccination strategies. The model, though relatively simple in structure, correctly describes the effect of vaccination, predicting the decrease in the number of varicella cases in post-vaccination period with reasonable accuracy. Further studies are needed to validate the predictions of epidemic models for other infectious diseases, including those transmitted by multiple pathogens, by non-direct contact or with various disease outcomes. By identifying areas for improvement and highlighting the model’s reliability within this context, the presented research provides insights for informing public health decision-making regarding infectious disease interventions.

## Figures and Tables

**Table 1:** The states of individuals considered in the model. Each compartment of the model corresponds to a specified state and a specified age.

State	Description
S	Susceptible, unvaccinated
E	Exposed (infected but not yet infectious for others), unvaccinated
I	Infectious, unvaccinated
R	Recovered, unvaccinated
SV1	Vaccinated with one dose and susceptible
SV2	Vaccinated with two doses and susceptible
VP1	Vaccinated with one dose and protected (immune)
VP2	Vaccinated with two doses and protected
EV	Vaccinated and exposed
IV	Vaccinated and infectious
RV	Vaccinated and recovered

## Data Availability

The original contributions presented in the study are included in the article; further inquiries can be directed to the corresponding author/s.

## Appendix A

### Appendix A.1. Deriving Prevalence from Seroprevalence, Including Maternal Protection

The estimation of disease prevalence from seroprevalence data was carried out in several steps. First, a smooth, non-decreasing function with values between 0 and 1 was fitted to the seroprevalence data (represented by dots in Figure 2c; data refer to ages 1 year and older). This was performed to enable a calculation of seroprevalence for all ages (empirical values were only reported for selected age groups). The modeled relationship between age a and seroprevalence π was represented by the curve in Figure 2c.

The function π(a) was then used to calculate the cumulative disease incidence in age groups (defined as the proportion of the population infected within these groups, as justified in Bollaerts et al. (2017) [39]). The cumulative incidence $\tilde{I}\left(a,a+∆a\right)$ for an age group between a and a+∆a is given by

(A1) $$\tilde{I}\left(a,a+∆a\right)=\pi \left(a+∆a\right)-\pi \left(a\right),$$

where $\pi \left(a\right)$ denotes seroprevalence at age a. Assuming that $\pi \left(0\right)=0$ and applying the above formula to age groups considered in the model allowed us to obtain ${\tilde{I}}_{i}$, the cumulative incidence for an age group between i and i+1 years old.

A sequence of cumulative incidences ${\tilde{I}}_{0}$, ${\tilde{I}}_{1}$, ${\tilde{I}}_{2}$, …, ${\tilde{I}}_{n-1}$ generated with the approach presented above does not take into account the effect of maternal protection, which is known to have an influence on varicella transmission (maternal antibodies acquired from seropositive mothers protect infants for several months after birth; seroprevalence at birth is non-zero) [56,61]. The model discussed in this work did not have a separate compartment for immune infants; maternal protection is considered in a simplified way. It is assumed that maternal protection only affects the first age group and that $\lambda_{0}$, the FOI (force of infection) in the first age group (0 to 1 year old), is the same as $\lambda_{1}$, the FOI in the next age group (1 to 2 years old). The difference in cumulative incidences is then due to the fact that the same FOI affects two groups with different proportions of susceptible individuals.

The reasoning presented below is used to obtain ${\tilde{I}}_{0}$ and ${\tilde{I}}_{1}$, the cumulative incidences for the first and for the second age group. For the remaining age groups (i=2, 3,…,n−1), the cumulative incidences were calculated directly from Equation (A1).

In equilibrium (i.e., when the number of new cases per unit time is constant), $\lambda_{i}$ can be expressed as

(A2) $$\lambda_{i}=\frac{∆I_{i}}{S_{i}∆t}=\frac{∆I_{i}/N_{i}}{S_{i}∆t/N_{i}},$$

where $∆I_{i}$ is the number of new disease cases in age group i at time ∆t, $S_{i}$ is the number of susceptible individuals in age group i, and $N_{i}$ is the number of all individuals in age group i. When ∆t is set to one year, the quantity $∆I_{i}/N_{i}$ becomes ${\tilde{I}}_{i}$, the cumulative incidence in the i-th age group, and one obtains

(A3) $$\lambda_{i}=\frac{{\tilde{I}}_{i}}{S_{i}/N_{i}}.$$

The proportion of susceptible individuals in the age group i can be estimated as the average value of $\left(1-\pi \left(a\right)\right)$ over the age group i:

(A4) $$\frac{S_{i}}{N_{i}}=\frac{1}{\left(i+1\right)-i}\left(1-\int_{i}^{i+1}\pi \left(a\right)da\right).$$

The value of $S_{0}/N_{0}$ was determined using the data on seroprevalence in infants under 1 year of age from Wutzler et al. (2001) [40], and the value of $S_{1}/N_{1}$ was determined from the fitted seroprevalence model π(a).

From the assumption that $\lambda_{0}=\lambda_{1},$ it follows that

(A5) $$\frac{{\tilde{I}}_{1}}{S_{1}/N_{1}}=\frac{{\tilde{I}}_{2}}{S_{2}/N_{2}}.$$

Furthermore, it is additionally assumed that the total cumulative incidence from the moment of birth to the age of 2 years is the same as if the seroprevalence at birth was equal to 0, i.e.,

(A6) $${\tilde{I}}_{0}+{\tilde{I}}_{1}=\pi \left(2\right).$$

In this way, the calculation of ${\tilde{I}}_{0}$ and ${\tilde{I}}_{1}$ remains consistent with the calculation of ${\tilde{I}}_{2}$, ${\tilde{I}}_{3}$, …, ${\tilde{I}}_{n-1}$ based on Equation (A1). In practice, the consideration of maternal protection in the manner presented here only modifies the ratio between the incidence (and, subsequently, the prevalence) in the first and in the second age groups. Consequently, ${\tilde{I}}_{0}$ and ${\tilde{I}}_{1}$ can be determined by using Equations (A5) and (A6). Once all the cumulative incidences ${\tilde{I}}_{0}$, ${\tilde{I}}_{1}$, ${\tilde{I}}_{2}$, …, ${\tilde{I}}_{n-1}$ had been obtained, the prevalence ${\hat{J}}_{i}$ for each age group i was calculated by using the following formula:

(A7) $${\hat{J}}_{i}={\tilde{I}}_{i}\cdot T_{infection},$$

where $T_{infection}\;$ is the average infection duration time. The resulting values of age-dependent prevalence, ${\hat{J}}_{i}$, were used in the calibration process.

### Appendix A.2. Model Equations

The evolution of the modeled system in continuous time (during a year) is driven by a set of differential equations. With compartments and parameters defined as in Table 1 and Table 2, N being the total number of individuals in the whole population, and the force of infection $\lambda_{i}$ in the i-th age group given by

(A8) $$\lambda_{i}=\omega +\frac{1}{N}\sum_{j=0}^{n-1}\alpha_{ij}c_{ij}\left(I_{j}+\xi \;IV_{j}\right),$$

and the equations are

(A9) $$\begin{array}{cc} \frac{dS_{i}}{dt}= & -\lambda_{i}S_{i}+\gamma_{R,S}R_{i}-\mu_{i}S_{i}\\ \frac{dE_{i}}{dt}= & \lambda_{i}S_{i}-\gamma_{E,I}E_{i}-\mu_{i}E_{i}\\ \frac{dI_{i}}{dt}= & \gamma_{E,I}E_{i}-\gamma_{I,R}I_{i}-\mu_{i}I_{i}\\ \frac{dR_{i}}{dt}= & \gamma_{I,R}I_{i}-\gamma_{R,S}R_{i}-\mu_{i}R_{i}\\ \frac{dSV_{i}}{dt}= & -\kappa \;\lambda_{i}SV_{i}+\gamma_{VP,SV}{VP}_{i}+\gamma_{R,S}RV_{i}-\mu_{i}{SV}_{i}\\ \frac{dVP_{i}}{dt}= & -\gamma_{VP,SV}{VP}_{i}-\mu_{i}{VP}_{i}\\ \frac{dEV_{i}}{dt}= & \kappa \;\lambda_{i}SV_{i}-\gamma_{E,I}EV_{i}-\mu_{i}{EV}_{i}\\ \frac{dIV_{i}}{dt}= & \gamma_{E,I}EV_{i}-\gamma_{I,R}IV_{i}-\mu_{i}{IV}_{i}. \end{array}$$

At the beginning of each year, an instant update of the age structure is performed:

- $S_{0}$ is assigned the value equal to the number of births $b\left(y\right)$ in the studied year y, and the remaining compartments corresponding to the first age group are set to 0.
- For i=1,2,…,n−1, each compartment pertaining to the age group i is assigned the value of the same compartment pertaining to the age group i−1.

Following the age structure update, vaccination-related compartments are updated (also instantaneously):

- From the $S_{a_{v1}}$ compartment (susceptible individuals at the age of first vaccine dose uptake), a fraction given by $g_{1}\left(y\right)$ (the coverage of the first vaccine dose in year y) is removed; the fraction of the removed individuals given by $\eta_{1}$ (the effectiveness of the first vaccine dose) is added to the $VP1_{a_{v1}}$ compartment, and the fraction of the removed individuals given by $1-\eta_{1}$ is added to the ${SV1}_{a_{v1}}$ compartment.
- From the ${SV}_{a_{v2}}$ compartment (susceptible individuals vaccinated with one dose, at the age of second vaccine dose uptake), a fraction given by $g_{2}\left(y\right)$ (the coverage of the second vaccine dose in year y) is removed; a fraction of the removed individuals given by $\eta_{2}$ (the effectiveness of the second vaccine dose) is added to the $VP2_{a_{v2}}$ compartment, and a fraction of the removed individuals given by $1-\eta_{1}$ is added to the ${SV2}_{a_{v2}}$ compartment.
- From the ${VP1}_{a_{v2}}$ compartment (individuals vaccinated and protected with one vaccine dose at the age of second dose uptake), a fraction given by $g_{2}\left(y\right)$ is moved into the ${VP2}_{a_{v2}}$ compartment. The form of this transition reflects the assumption that the vaccine’s second dose has the efficacy of 100% for the individuals who are successfully vaccinated with the first dose.

The presented components of the modeled system’s evolution (age structure update, vaccination, and differential equations) are repeated in annual cycles.

### Appendix A.3. Calibration

Since the parameters $\alpha_{ij}$, used to specify the values of the force of infection $\lambda_{i}$ given by Equation (A8), are not directly observed, they were calibrated such that the resulting force of infection minimizes the difference between the predicted the and observed prevalence of varicella in pre-vaccination era.

The parameters $\alpha_{ij}$ represent the risk of virus transmission from an infectious individual of age j  to a susceptible individual of age i. The risk of virus transmission between them may depend on both ages and various formulations for the transmission rate were considered in the literature [31,32,52,62]. The most biologically plausible assumption was made that $\alpha_{ij}$ depends only on the age of the susceptible individual i involved in the contact (i.e., $\alpha_{ij}=\alpha_{i}$).

To calibrate $\alpha_{ij}$, the modeled system was considered at equilibrium. Discrete events, such as births and aging, occur in the model once a year, causing disruptions of the system and resulting in noncontinuous behavior. With these events considered, equilibrium could never be reached. Therefore, to enable the calibration of $\alpha_{ij}$, the model equations were adjusted in such a way that births and aging were implemented as continuous flows between age groups. This adjustment allows for the calibration of $\alpha_{ij}$ and obtaining the corresponding steady state. The adjusted equations are slightly different from the original ones; therefore, the precision of the calibration procedure is inherently limited.

The system of equations for which steady state is sought in the calibration process is given below. Since the calibration pertains to the pre-vaccination era, only the compartments corresponding to natural varicella (S, E, I, and R) were taken into account.

(A10) $$\mathrm{Equations}\;\mathrm{for}\;\mathrm{the}\;\mathrm{first}\;\mathrm{age}\;\mathrm{group}\;(i=0):\;\;\;\;\;\;\;\;\;\;\;\;\;\;\;\;\;\;\;\;\;\;\;\;\;\;\;\;\;\;\;\;\;\;\;\;\;\;\;\;\;\;\;\;\;\;\;\;\;\;\;\;\;\;\;\;\;\;\;\;\;\;\;\;\;\;\;\;\;\;\;\;\;\frac{dS_{i}}{dt}=\frac{b(0)}{T_{ac}}+\gamma_{R,S}R_{i}-\frac{S_{i}}{T_{ac}}-\lambda_{i}S_{i}-\mu_{i}S_{i}\;\frac{dE_{i}}{dt}=\lambda_{i}S_{i}-\frac{E_{i}}{T_{ac}}-\gamma_{E,I}E_{i}-\mu_{i}E_{i}\;\frac{dI_{i}}{dt}=\gamma_{E,I}E_{i}-\frac{I_{i}}{T_{ac}}-\gamma_{I,R}I_{i}-\mu_{i}I_{i}\;\frac{dR_{i}}{dt}=\gamma_{I,R}I_{i}-\frac{R_{i}}{T_{ac}}-\gamma_{R,S}R_{i}-\mu_{i}R_{i}.\;\mathrm{Equations}\;\mathrm{for}\;\mathrm{the}\;\mathrm{remaining}\;\mathrm{age}\;\mathrm{groups}\;(i>0):\;\;\;\;\;\;\;\;\;\;\;\;\;\;\;\;\;\;\;\;\;\;\;\;\;\;\;\;\;\;\;\;\;\;\;\;\;\;\;\;\;\;\;\;\;\;\;\;\;\;\;\;\;\;\;\;\;\;\;\;\;\;\;\;\;\;\;\;\;\;\;\;\;\frac{dS_{i}}{dt}=\frac{S_{i-1}}{T_{ac}}+\gamma_{R,S}R_{i}-\frac{S_{i}}{T_{ac}}-\lambda_{i}S_{i}-\mu_{i}S_{i}\;\frac{dE_{i}}{dt}=\frac{E_{i-1}}{T_{ac}}+\lambda_{i}S_{i}-\frac{E_{i}}{T_{ac}}-\gamma_{E,I}E_{i}-\mu_{i}E_{i}\;\frac{dI_{i}}{dt}=\frac{I_{i-1}}{T_{ac}}+\gamma_{E,I}E_{i}-\frac{I_{i}}{T_{ac}}-\gamma_{I,R}I_{i}-\mu_{i}I_{i}\;\frac{dR_{i}}{dt}=\frac{R_{i-1}}{T_{ac}}+\gamma_{I,R}I_{i}-\frac{R_{i}}{T_{ac}}-\gamma_{R,S}R_{i}-\mu_{i}R_{i}.$$

In the above equations, $b\left(0\right)$ was the number of births in the last year before the introduction of vaccination (the last year of the pre-vaccination era), and $T_{ac}$ was the time corresponding to a single aging cycle (1 year).

At equilibrium, $S_{i}$, $E_{i}$, $I_{i}$, $R_{i}$ are constant, and thus the derivatives on the left-hand side of each of the Equations (A10) are equal to 0. The values of the force of infection, expressed in the pre-vaccination era by

(A11) $$\lambda_{i}=\omega +\frac{1}{N}\sum_{j=0}^{n-1}\alpha_{ij}c_{ij}I_{j},$$

are also constant.

Calibration involves determining the values of $\alpha_{ij}$ so that the age-dependent force of infection rates $\lambda_{0},\lambda_{1},\ldots ,\lambda_{n-1}$ computed with these values, along with the estimated contact matrix and age-specific varicella incidence, when inserted into Equations (A10), results in a steady state where the disease prevalence rates $I_{i}/N_{i}$ ($N_{i}$ denotes the number of individuals in age group i) minimize the distance from the observed prevalence ${\hat{J}}_{i}$ over all age groups. The distance between $I_{i}/N_{i}$ and ${\hat{J}}_{i}$ was measured by the sum of squared residuals. In the calibration process, it was minimized numerically, using the differential evolution algorithm.

### Appendix A.4. Social Contact Matrix

In the data on social contact patterns reported in Mossong et al. (2008) [37], the population was divided into 15 age groups: 0–5, 6–10, 11–15, …, 61–65, 66–70, 71–100 (these groups are here indexed by indices with primes—i’, j’). The data allowed us to determine the quantity $m_{i’j’}$, defined as the average number of contacts per unit time that a single individual from group j’ has with individuals from group i’. Based on that, the total number of contacts between groups i’ and j’ per unit time can be calculated as $m_{i’j’}N_{j’}$, where $N_{j’}$ is the number of individuals in group j’. Since the data reported in Mossong et al. (2008) was collected via surveys and, therefore, do not guarantee balance, the numbers of contacts between groups i’ and j’ per unit time computed in two ways—as $m_{i’j’}N_{j’}$ and as $m_{j’i’}N_{i’}$—are not guaranteed to be equal. To eliminate these differences, the corrected total number of contacts per unit time between groups i’ and j’, denoted by $K_{i’j’}$, was estimated as the arithmetic mean of the two values [63]:

(A12) $$K_{i^{’}j^{’}}=\frac{m_{i^{’}j^{’}}N_{j^{’}}+m_{j^{’}i^{’}}N_{i^{’}}}{2}.$$

The age grouping used in the model (indexed by indices without primes) had a greater resolution than that used in Mossong et al. (2008) (each age group in the model is a subset of some age group considered in Mossong et al. (2008) [37]). To determine $K_{ij}$, which represents the total number of contacts between model-defined groups i and j per unit time, the following assumption was made: the contribution to $K_{i^{’}j^{’}}$ from $K_{ij}$ is proportional to $N_{i}N_{j}$, the product of the sizes of groups i and j:

(A13) $$K_{ij}=\frac{N_{i}N_{j}}{N_{i^{’}}N_{j^{’}}}K_{i^{’}j^{’}}$$

With $K_{ij}$ computed, the elements of the contact matrix $c_{ij}$ used in the model can be computed using the following relationship:

(A14) $$c_{ij}=\frac{K_{ij}N}{N_{i}N_{j}},$$

where N denotes the total number of individuals in the whole population, $N=\sum_{i=0}^{n-1}N_{i}$.
